# Supplementary material for: Effect of Ginseng Extracts on the Improvement of Osteopathic and Arthritis Symptoms in Women with Osteopenia: A Randomized, Double-Blind, Placebo-Controlled Clinical Trial
Source: Nutrients. 2021 Sep 24;13(10):3352. doi: 10.3390/nu13103352 (PMC8539988; doi:10.3390/nu13103352)
Supplement: Supplementary file 1 [file nutrients-13-03352-s001.zip › nutrients-1373217-supplementary.pdf]

Supplementary Table S1. Laboratory profiles of the participants

Laboratory profiles (standard range)	GE Low(1g) group (n=30)		GE High(3g) group (n=30)		Placebo group (n=30)		
	Week 0	Week 12	Week 0	Week 12	Week 0	Week 12	p-value[1]
WBC (4.8–10.8×10^3 /μL)	5.30±1.72	5.08±1.42	5.07±1.12	4.83±1.04	4.94±1.03	4.70±0.83	0.951
RBC (4.2–5.4×100^3 /μL)	4.35±0.32	4.32±0.34	4.41±0.30	4.42±0.29	4.40±0.33	4.41±0.27	0.825
Hemoglobin (12–16 g/dL)	13.24±0.89	13.09±0.82	13.31±0.76	13.30±0.81	13.34±0.86	13.38±0.78	0.539
Hematocrit (37–47%)	39.08±2.37	39.52±2.26	39.37±39.96	39.96±2.11	39.59±2.42	40.27±2.13	0.947
Platelet (130–450×10^3 /μL)	262.47±46.60	266.03±44.10	255.57±51.49	263.27±55.09	249.43±54.92	255.81±61.72	0.800
ALP (8–48 IU/L)	79.73±14.95	81.24±16.22	75.33±16.45	78.43±16.67	86.63±21.45	86.19±19.53	0.302
GGT (8–48 IU/L)	18.90±10.91	20.24±13.37	18.23±10.96	17.53±10.92	19.47±9.99	1.07±11.80	0.372
AST (12–33 IU/L)	23.77±4.97	23.34±5.72	24.00±4.90	22.67±3.30	24.07±4.98	24.85±5.16	0.258
ALT (5–35 IU/L)	24.50±10.46	23.45±12.41	23.97±10.10	21.60±4.87	23.10±8.14	23.70±7.52	0.400
Total bilirubin (0.2–1.2 mg/dL)	0.81±0.25	0.77±0.17	0.74±0.17	0.71±0.15	0.82±0.28	0.80±0.16	0.973
Total protein (6.7–8.3 g/dL)	7.26±0.30	7.38±0.28	7.30±0.22	7.51±0.26	7.22±0.34	7.33±0.39	0.324
Albumin (3.5–5.3 g/dL)	4.29±0.8	4.29±0.14	4.36±0.18	4.36±0.18	4.32±0.18	4.31±0.17	0.977
BUN (8–23 mg/dL)	13.87±2.71	14.90±3.95	14.07±2.92	14.33±3.02	13.83±3.96	13.70±2.766	0.394
Creatinine (0.7–1.7 mg/dL)	0.60±0.09	0.58±0.08	0.56±0.10	0.55±0.09	0.56±0.08	0.55±0.09	0.918
Total cholesterol (mg/Dl)	205.87±25.38	211.21±25.09	199.97±33.52	210.53±37.41	209.97±34.30	211.70±38.29	0.198
Triglyceride (mg/dL)	121.40±36.57	137.69±77.78	129.7±73.85	110.93±45.23	122.47±79.64	119.67±70.16	0.065
HDL-cholesterol (mg/dL)	55.70±13.38	56.86±14.95	52.27±11.40	54.00±11.10	58.90±15.70	58.41±15.01	0.309
LDL-cholesterol (mg/dL)	125.89±25.56	126.81±25.02	121.71±30.86	134.35±33.30	126.57±27.86	129.36±30.71	0.050
Glucose (74–106 mg/dL)	85.43±6.94	85.69±8.50	84.80±6.39	85.50±7.44	87.00±7.94	85.22±7.56	0.370
Estradiol (pg/mL)	83.46±32.65	78.55±23.98	79.99±27.59	76.63±26.90	83.67±29.94	79.61±32.26	0.386

FSH (mIL/Ml)	374.50±45.77	395.03±50.78	391.10±51.84	406.987±69.75	392.03±48.68	403.26±52.03	0.932
LDH (IU/L)	9.79±23.86	6.66±6.34	6.18±2.92	5.64±1.85	8.11±12.18	13.65±32.07	0.838
Creatinine Kinase (IU/L)	86.07±29.90	103.59±58.95	96.23±49.60	102.70±50.97	91.70±37.95	128.00±91.64	0.217
Specific Gravity	1.02±0.00	1.02±0.01	1.02±0.00	1.02±0.01	1.02±0.01	1.02±0.01	0.974
pH	5.85±0.80	6.16±0.85	5.93±0.96	5.97±0.92	6.27±0.80	6.33±0.93	0.369

Data are presented as mean ± SD.

[1]Analyzed by linear mixed model.

Abbreviations: GE, Ginseng Extract; WBC,White Blood Cell; RBC, Red Blood Cell; ALP; Alkaline Phosphatase; GGT, Gamma Glutamyl Transferase; AST;Aspartate Transaminase; ALT,Alanine Transaminase; BUN, Blood Urea Nitrogen; CK, creatine kinase; LDH, lactate dehydrogenase, FSH, follicle stimulating hormone.
